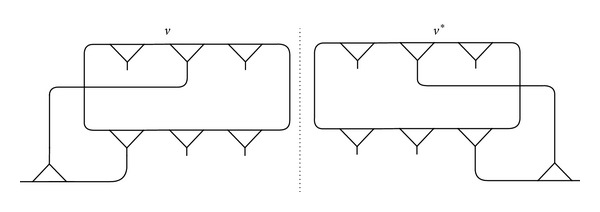# Equivalent Pairs of Words and Points of Connection

**DOI:** 10.1155/2014/505496

**Published:** 2014-09-08

**Authors:** Qaiser Mushtaq, Abdul Razaq

**Affiliations:** Department of Mathematics, Quaid-i-Azam University, Islamabad, Pakistan

## Abstract

Higman has defined coset diagrams for PGL(2, ℤ). The coset diagrams are composed of fragments, and the fragments are further composed of two or more circuits. A condition for the existence of a certain fragment *γ* in a coset diagram is a polynomial *f* in ℤ[𝔃], obtained by choosing a pair of words *F*[*w*_*i*_, *w*_*j*_] such that both *w*_*i*_ and *w*_*j*_ fix a vertex *v* in *γ*. Two pairs of words are equivalent if and only if they have the same polynomial. In this paper, we find distinct pairs of words that are equivalent. We also show there are certain fragments, which have the same orientations as those of their mirror images.

## 1. Introduction

The modular group PSL(2, *Z*) [1–3] an interesting group of hyperbolic isometries and has a finite presentation

(1) $$\begin{array}{c} \langle x,y:x^{2}=y^{3}=1\rangle , \end{array}$$

where *x* and *y* are the linear fractional transformations defined by *z* → −1/*z* and *z* → (*z* − 1)/*z*, respectively. The modular group PSL(2, *Z*) is of great interest in many fields of mathematics, for example, group theory, graph theory, and number theory. By adjoining a new element *t* : *z* → 1/*z* with *x* and *y*, we obtain a presentation

(2) $$\begin{array}{c} \langle x,y,t:x^{2}=y^{3}=t^{2}={\left(xt\right)}^{2}={\left(yt\right)}^{2}=1\rangle \end{array}$$

of the extended modular group PGL(2, *Z*).

The Hecke group *H*(*λ*_*q*_) is the discrete group generated by *z* → −1/*z*,  *z* → −1/(*z* + *λ*_*q*_), where *λ*_*q*_ = 2cos⁡(*π*/*q*). It is well known that the modular group can be generalized to the Hecke groups. For *q* = 3, the resulting Hecke group *H*(*λ*_3_) is the modular group PSL(2, *Z*).

If *q* is a power of a prime *p*, then the projective line over the finite field *F*_*q*_, which is *F*_*q*_ ∪ {*∞*}, is denoted by PL(*F*_*q*_). The group PGL(2, *q*) has its customary meaning, a group of all linear fractional transformations *z* → (*az* + *b*)/(*cz* + *d*) such that *a*, *b*, *c*, *d* ∈ *F*_*q*_ and *ad* − *bc* ≠ 0, while PSL(2, *q*) is its subgroup consisting of all those, where *ad* − *bc* is a non-zero square in *F*_*q*_.

There is a well-known relationship between the action of PSL(2, *Z*) on *R* and continued fractions. There are many articles on the connection between the geodesics on the modular surface and continued fractions which are particularly important in the theory of approximation of real numbers by rational [4, 5]. Important results have been obtained using these ideas in a very well-written article [6]. A good account on relationship between continued fractions and indefinite binary quadratic forms is also given in [7]. In [8], Mushtaq has shown a relationship between reduced indefinite binary quadratic forms and orbits of the modular group. There is another interesting article [9] of Mushtaq and Hayat on the topic of Pell numbers, Pell-Lucas numbers, and modular group. In [10], Bong and Mushtaq determine the Fibonacci and Locus numbers through the action of modular group on real quadratic fields.

In 1978, G. Higman introduced a new type of graph called a coset diagram for PGL(2, *Z*). The three cycles of *y* are denoted by small triangles whose vertices are permuted counterclockwise by *y* and any two vertices which are interchanged by *x* are joined by an edge. The fixed points of *x* and *y* are denoted by heavy dots. Notice (*yt*)^2^ = 1 is equivalent to *t* *yt* = *y* ^−1^, which means that *t* reverses the orientation of the triangles representing the three cycles of *y* (as reflection does); because of this, there is no need to make the diagram more complicated by introducing *t*-edges. For details about coset diagrams, one can refer to [11–14].

Two homomorphisms *α* and *β* from PGL(2, *Z*) to PGL(2, *q*) are called conjugate if *β* = *αρ* for some inner automorphism *ρ* on PGL(2, *q*). We call *α* to be non-degenerate if neither of *x*, *y* lies in the kernel of *α*. In [15], it has been shown that the conjugacy classes of non-degenerate homomorphisms from PGL(2, *Z*) to PGL(2, *q*) are in one to one correspondence with the elements *θ* ≠ 0,3 of *F*_*q*_ under the correspondence which maps each class to its parameter *θ*. As in [15], the coset diagram corresponding to the action of PGL(2, *Z*) on PL(*F*_*q*_) via a homomorphism *α* with parameter *θ* is denoted by *D*(*θ*, *q*). Each conjugacy class is represented by a unique coset diagram, unique in the sense that the diagram remains invariant except that labels of vertices change from *α* to *β*.

By a circuit in a coset diagram for an action of PGL(2, *Z*) on PL(*F*_*q*_), we will mean a closed path of triangles and edges. Let *n*_1_, *n*_2_,…, *n*_2*k*_ be a sequence of positive integers, the circuit which contains a vertex, fixed by *w* = (*xy*)^*n*_1_^(*xy* ^−1^)^*n*_2_^ ⋯ (*xy* ^−1^)^*n*_2*k*_^ ∈ PSL(2, *Z*) for some *k* ≥ 1; we will mean the circuit in which *n*_1_ triangles have one vertex inside the circuit and *n*_2_ triangles have one vertex outside the circuit and so on. Since it is a cycle (*n*_1_, *n*_2_,…, *n*_2*k*_), so it does not make any difference if *n*_1_ triangles have one vertex outside the circuit and *n*_2_ triangles have one vertex inside the circuit and so on. The circuit of the type (*n*_1_, *n*_2_,…, *n*_2*k*′_, *n*_1_, *n*_2_,…, *n*_2*k*′_,…, *n*_1_, *n*_2_,…, *n*_2*k*′_) is called a periodic circuit and the length of its period is 2*k*′. A circuit that is not of this type is non-periodic. A circuit is called simple, if each vertex of the circuit is fixed by a unique word *w* or its inverse *w* ^−1^.

## 2. Joining of Circuits

Consider two non-periodic and simple circuits (*n*_1_, *n*_2_,…, *n*_2*k*_) and (*m*_1_, *m*_2_,…, *m*_2*k*_). Let *v*_*i*_ be any vertex in (*n*_1_, *n*_2_,…, *n*_2*k*_) fixed by a word *w*_*i*_ and let *v*_*j*_ be any vertex in (*m*_1_, *m*_2_,…, *m*_2*k*_) fixed by a word *w*_*j*_. In order to connect these two circuits at *v*_*i*_ and *v*_*j*_, we choose, without loss of generality (*n*_1_, *n*_2_,…, *n*_2*k*_), and apply *w*_*j*_ on *v*_*i*_ in such a way that *w*_*j*_ ends at *v*_*i*_. Consequently, we get a fragment denoted by *γ*. In other words, by *γ*, we will mean a nonsimple fragment whose one vertex *v* = *v*_*i*_ = *v*_*j*_ is fixed by a pair of words *w*_*i*_, *w*_*j*_, dented by *F*[*w*_*i*_, *w*_*j*_].

**Example 1 .**

**Figure 1:**
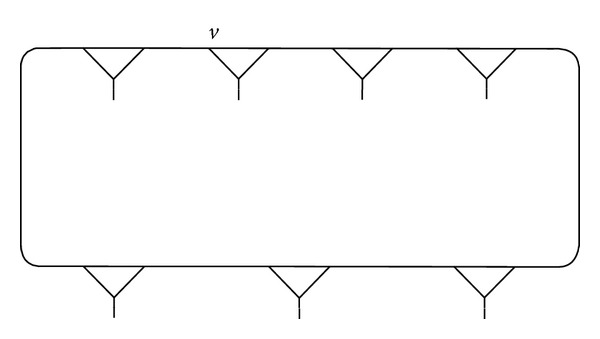


**Figure 2:**
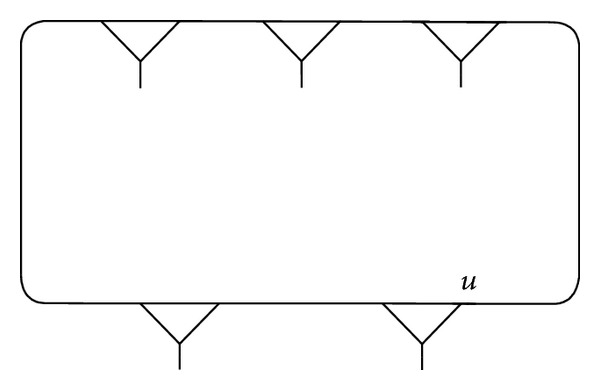


**Figure 3:**
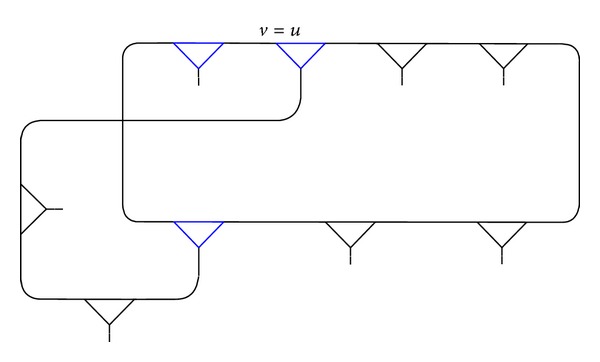


We connect the vertex *v*, fixed by (*xy*)(*xy* ^−1^)^3^(*xy*)^3^, in (4,3) with the vertex *u*, fixed by (*xy*)^3^(*xy* ^−1^)^2^ of (3,2), and compose a fragment *γ* as shown in Figures 1, 2, and 3.

One can see that the vertex *v* = *u* in *γ* (Figure 3) is fixed by a pair of words

(3) $$\begin{array}{c} F\left[\left(xy\right){\left(xy^{-1}\right)}^{3}{\left(xy\right)}^{3},{\left(xy\right)}^{3}{\left(xy^{-1}\right)}^{2}\right]. \end{array}$$

The action of PGL(2, *Z*) on PL(*F*_*q*^2^_) yields two components, namely, PL(*F*_*q*_) and PL(*F*_*q*^2^_)∖PL(*F*_*q*_). For sake of simplicity, let $\bar{\text{PL}(F_{q})}$ denote the complement PL(*F*_*q*^2^_)∖PL(*F*_*q*_).

The coset diagram *D*(*θ*, *q*) is made of fragments. It is therefore necessary to ask when a fragment exists in *D*(*θ*, *q*). In [16], this question is answered in the following way.

**Theorem 2 .**

Given a fragment, there is a polynomial *f* in *Z*[*z*] such that

- if the fragment occurs in *D*(*θ*, *q*), then *f*(*θ*) = 0;
- if *f*(*θ*) = 0, then the fragment, or a homomorphic image of it occurs in *D*(*θ*, *q*) or in $\bar{PL(F_{q})}$.

In [16], the method of calculating a polynomial from a fragment is given. Here, we describe this method briefly. Since a fragment is composed of two non-periodic and connected circuits (*n*_1_, *n*_2_,…, *n*_2*k*_) and (*m*_1_, *m*_2_,…, *m*_2*k*_) with a common fixed vertex, say, *v*, then there is a pair of words, for instance,

(4) F[wi,wj]=F[(xy)l1(xy−1)l2⋯(xy−1)l2k1,   (xy)m1(xy−1)m2⋯(xy−1)m2k2]

such that (*v*)*w*_*i*_ = *v* and (*v*)*w*_*j*_ = *v*. Let *X* and *Y* be the matrices corresponding to *x* and *y* of PGL(2, *q*). Then, *w*_*i*_ and *w*_*j*_ can be expressed as

(5) $$\begin{array}{c} W_{i}={\left(XY\right)}^{l_{1}}{\left(XY^{-1}\right)}^{l_{2}}\cdots {\left(XY^{-1}\right)}^{l_{2k_{1}}},\\ W_{j}={\left(XY\right)}^{m_{1}}{\left(XY^{-1}\right)}^{m_{2}}\cdots {\left(XY^{-1}\right)}^{m_{2k_{2}}}, \end{array}$$

where *k*_1_, *k*_2_ > 0. By using (3.1) to (3.7) of [16], the matrices *W*_*i*_ and *W*_*j*_ can be expressed linearly as

(6) $$\begin{array}{c} W_{i}=\lambda_{0}I+\lambda_{1}X+\lambda_{2}Y+\lambda_{3}XY,\\ W_{j}=\mu_{0}I+\mu_{1}X+\mu_{2}Y+\mu_{3}XY \end{array}$$

such that *λ*_*i*_ and *μ*_*i*_, for *i* = 0,1, 2,3, are polynomials in *r* and Δ, where *r* is the trace of *XY* and Δ is its determinant. Since *v* is a common fixed vertex of *w*_*i*_ and *w*_*j*_, therefore the 2 × 2 matrices *W*_*i*_ and *W*_*j*_ have a common eigenvector. Then, by Lemma 3.1 of [16], the algebra generated by *W*_*i*_ and *W*_*j*_ has dimension 3. The algebra contains *I*, *W*_*i*_, *W*_*j*_, and  *W*_*i*_ *W*_*j*_ and so these are linearly dependent. Using (3.1) to (3.7) of [16], the matrix *W*_*i*_ *W*_*j*_ is expressed as

(7) $$\begin{array}{c} W_{i}W_{j}=\nu_{0}I+\nu_{1}X+\nu_{2}Y+\nu_{3}XY, \end{array}$$

where *v*_*i*_, for *i* = 0,1, 2,3, can be calculated in terms of the *λ*_*i*_ and *μ*_*i*_, using (3.1) to (3.7) of [16]. The condition that *I*, *W*_*i*_, *W*_*j*_, and *W*_*i*_ *W*_*j*_ are linearly dependent is expressed as

(8) $$\begin{array}{c} \left|\begin{array}{ccc} \lambda_{1} & \lambda_{2} & \lambda_{3}\\ \mu_{1} & \mu_{2} & \mu_{3}\\ \nu_{1} & \nu_{2} & \nu_{3} \end{array}\right|=0. \end{array}$$

If we calculate *v*_1_, *v*_2_, *v*_3_ in terms of *λ*_*i*_ and *μ*_*i*_ and substitute in (8), we find that it is equivalent to

(9) (λ2μ3−μ2λ3)2+Δ(λ3μ1−μ3λ1)2+(λ1μ2−μ1λ2)2  +r(λ2μ3−μ2λ3)(λ3μ1−μ3λ1)  +(λ2μ3−μ2λ3)(λ1μ2−μ1λ2)=0.

This gives a homogeneous equation in Δ and *r*. By substituting Δ*θ* for *r* ^2^, we get an equation in *θ*.

Two pairs of words *F*[*w*_*i*_, *w*_*j*_] and *F*[*w*_*k*_, *w*_*l*_] are equivalent if and only if they have the same polynomial. If two pairs of words *F*[*w*_*i*_, *w*_*j*_] and *F*[*w*_*k*_, *w*_*l*_] are equivalent, then we write *F*[*w*_*i*_, *w*_*j*_] ~ *F*[*w*_*k*_, *w*_*l*_].

In this paper, we find distinct pairs of words that are equivalent. We also show there are certain fragments, which have the same orientations as those of their mirror images.

## 3. Main Results

**Theorem 3 .**

In the above notation, the polynomial obtained from a fragment *γ* is unique.

**Proof**

Let *v*_*i*_ and *v*_*k*_ be any two vertices of a fragment *γ*, such that *v*_*i*_ is fixed by a pair of words *F*[*w*_*i*_, *w*_*j*_] and *v*_*k*_ is fixed by a pair of words *F*[*w*_*k*_, *w*_*l*_]. Suppose *f*(*θ*) is a polynomial obtained by choosing *F*[*w*_*i*_, *w*_*j*_] and *g*(*θ*) is a polynomial obtained by choosing *F*[*w*_*k*_, *w*_*l*_]. Now, if *f*(*θ*) = 0, then, by Theorem 2, the fragment *γ* or its homomorphic image occurs in *D*(*θ*, *q*) or in $\bar{\text{PL}(F_{q})}$. So, there exists a vertex in *D*(*θ*, *q*) or in $\bar{\text{PL}(F_{q})}$ which is fixed by *F*[*w*_*k*_, *w*_*l*_]. Again, by Theorem 2, we have *g*(*θ*) = 0. Similarly, it can be proved that if *g*(*θ*) = 0, then *f*(*θ*) = 0. Hence, *f*(*θ*) = *g*(*θ*).

**Remark 4 .**

If a vertex *v* is fixed by a word *w*_*i*_ ∈ PSL(2, *Z*), then the vertex (*v*)*w* is fixed by the conjugate *w* ^−1^ *w*_*i*_ *w* of *w*_*i*_.

We know that *γ* is a fragment created by joining a vertex *v*_*i*_ of (*n*_1_, *n*_2_,…, *n*_2*k*_) with the vertex *v*_*j*_ of (*m*_1_, *m*_2_,…, *m*_2*k*_). Since the same polynomial is evolved for all the points of connection for the fragment *γ*, therefore it is important to know all the points of connection for the fragment *γ*. Following theorem is useful in finding all points of connection of (*n*_1_, *n*_2_,…, *n*_2*k*_) and (*m*_1_, *m*_2_,…, *m*_2*k*_) for the fragment *γ*.

**Theorem 5 .**

Let fragment *γ* be constructed by joining a vertex *v*_*i*_ of (*n*_1_, *n*_2_,…, *n*_2*k*_) with the vertex *v*_*j*_ of (*m*_1_, *m*_2_,…, *m*_2*k*_). Then, *γ* is obtainable also, if the vertex (*v*_*i*_)*w* of (*n*_1_, *n*_2_,…, *n*_2*k*_) is joined with the vertex (*v*_*j*_)*w* of (*m*_1_, *m*_2_,…, *m*_2*k*_).

**Proof**

Suppose vertex *v*_*i*_ is fixed by a word *w*_*i*_ and vertex *v*_*j*_ is fixed by a word *w*_*j*_. Then, one vertex of the fragment *γ* is fixed by *w*_*i*_ and *w*_*j*_. Now, we join the vertex (*v*_*i*_)*w* of (*n*_1_, *n*_2_,…, *n*_2*k*_) with the vertex (*v*_*j*_)*w* of (*m*_1_, *m*_2_,…, *m*_2*k*_) and compose a fragment *δ*. By Remark 4, the vertex (*v*)*w* of fragment *δ* is fixed by *w* ^−1^ *w*_*i*_ *w* and *w* ^−1^ *w*_*j*_ *w*, whereas the vertex ((*v*)*w*)*w* ^−1^ = *v* of fragment *δ* is fixed by *w*(*w* ^−1^ *w*_*i*_ *w*)*w* ^−1^ = *w*_*i*_ and *w*(*w* ^−1^ *w*_*j*_ *w*)*w* ^−1^ = *w*_*j*_. This shows that *γ* and *δ* are the same fragments.

Since *γ* and *δ* are the same fragments, and a unique polynomial is obtained from a fragment, hence *F*[*w*_*i*_, *w*_*j*_] ~ *F*[*w* ^−1^ *w*_*i*_ *w*, *w* ^−1^ *w*_*j*_ *w*].

**Corollary 6 .**

Let *P* be the set of words such that for any *w* ∈ *P*, both vertices (*v*_*i*_)*w* and (*v*_*j*_)*w* lie on the circuits (*n*_1_, *n*_2_,…, *n*_2*k*_) and (*m*_1_, *m*_2_,…, *m*_2*k*_). Let *s* be the total number of points of connection of the circuits to compose *γ*. Then, *s* = |*P*|.

**Example 7 .**

As in Example 1, the vertex *v* in (4,3) is connected with the vertex *u* in (3,2), and a fragment *γ* is evolved. Then, one can see that

(10) $$\begin{array}{c} P=\left\{y,y^{-1},e,x,xy,xy^{-1},xyx,xyxy,xyxy^{-1}\right\} \end{array}$$

is the set of words such that, for any *w* ∈ *P*, both vertices (*v*) *w* and (*u*) *w* lie on (4,3) and (3,2). Since |*P* | = 9, there are nine points of connection of (4,3) and (3,2) composing *γ*. Hence, 9 vertices of one circuit are merged in another circuit. In other words, 3 triangles of one circuit are merged in another circuit.

The number of points of connection of the circuits for a fragment is always multiple of three and plays a significant role because they are directly related to the structure of the fragment. The following theorem illustrates this relation.

**Theorem 8 .**

Let *r* be the total number of triangles in a fragment *γ* and *s* the total number of points of connection of the circuits (*n*_1_, *n*_2_,…, *n*_2*k*_) and (*m*_1_, *m*_2_,…, *m*_2*k*_). Then, *r* = ∑_*j*=1_^2*k*^ *n*_*j*_ + ∑_*j*=1_^2*k*^ *m*_*j*_ − (1/3)*s*.

**Proof**

Since circuits (*n*_1_, *n*_2_,…, *n*_2*k*_) and (*m*_1_, *m*_2_,…, *m*_2*k*_) have ∑_*j*=1_^2*k*^ *n*_*j*_ and ∑_*j*=1_^2*k*^ *m*_*j*_ number of triangles, respectively, and there are *s* number of points of connection of (*n*_1_, *n*_2_,…, *n*_2*k*_) and (*m*_1_, *m*_2_,…, *m*_2*k*_) composing *γ*, therefore, by Corollary 6, there are *s* number of words *w* such that (*v*_*i*_) *w*, (*v*_*j*_) *w* lie on (*n*_1_, *n*_2_,…, *n*_2*k*_) and (*m*_1_, *m*_2_,…, *m*_2*k*_), respectively. So, *s* number of vertices of (*m*_1_, *m*_2_,…, *m*_2*k*_) are merged in (*n*_1_, *n*_2_,…, *n*_2*k*_), and remaining 3∑_*j*=1_^2*k*^ *m*_*j*_ − *s* number of vertices are included in (*n*_1_, *n*_2_,…, *n*_2*k*_) to compose *γ*. So, the total number of vertices in *γ* is (3∑_*j*=1_^2*k*^ *m*_*j*_ − *s* + 3∑_*j*=1_^2*k*^ *n*_*j*_). This implies that *r* = ∑_*j*=1_^2*k*^ *n*_*j*_ + ∑_*j*=1_^2*k*^ *m*_*j*_ − (1/3)*s*.

**Remark 9 .**

Consider a fragment *γ* such that one vertex *v* of *γ* is fixed by *F*[*w*_*i*_, *w*_*j*_]. Then, obviously, *v* is fixed by *F*[*w*_*i*_^−1^, *w*_*j*_^−1^]. So, *F*[*w*_*i*_, *w*_*j*_] ~ *F*[*w*_*i*_^−1^, *w*_*j*_^−1^]. Also, it is trivial that *F*[*w*_*i*_, *w*_*j*_] ~ *F*[*w*_*j*_, *w*_*i*_].

The following Theorem shows that (*n*_1_, *n*_2_,…, *n*_2*k*_) and (*m*_1_, *m*_2_,…, *m*_2*k*_) are not a unique pair of circuits for the fragment *γ*.

**Theorem 10 .**

The following pairs of words are equivalent:

(11) $$\begin{array}{c} F\left[w_{i},w_{j}\right],\;\;F\left[w_{i},w_{i}w_{j}\right],\;\;F\left[w_{i},w_{i}w_{j}^{-1}\right],\\ F\left[w_{i},w_{j}w_{i}\right],\;\;F\left[w_{i},w_{j}w_{i}^{-1}\right]. \end{array}$$

**Proof**

Let *v*_*i*_, *v*_*j*_, and *v*_*k*_ be arbitrary vertices of the circuits (*n*_1_, *n*_2_,…, *n*_2*k*_), (*m*_1_, *m*_2_,…, *m*_2*k*_), and (*m*_1_′, *m*_2_′,…, *m*_2*k*_′), respectively. Let the words *w*_*i*_, *w*_*j*_, and *w*_*i*_ *w*_*j*_ fix the vertices *v*_*i*_, *v*_*j*_, and *v*_*k*_, respectively. We join a vertex *v*_*i*_ fixed by *w*_*i*_ of the circuit (*n*_1_, *n*_2_,…, *n*_2*k*_) with the vertex *v*_*j*_ fixed by *w*_*j*_ of the circuit (*m*_1_, *m*_2_,…, *m*_2*k*_) to compose *γ*. Also, we join a vertex *v*_*i*_ fixed by *w*_*i*_ of the circuit (*n*_1_, *n*_2_,…, *n*_2*k*_) with the vertex fixed by *w*_*i*_ *w*_*j*_ of the (*m*_1_′, *m*_2_′,…, *m*_2*k*_′) to compose a fragment *δ*. So, in *δ*, *v*_*i*_ is a common fixed vertex of *F*[*w*_*i*_, *w*_*i*_ *w*_*j*_]. The fragment *δ* is different from the fragment *γ* if and only if, in fragment *δ*, the word *w*_*i*_ *w*_*j*_ fixes the vertex *v*_*i*_ in the following way: *w*_*i*_ maps the vertex *v*_*i*_ to some vertex *a* ≠ *v*_*i*_, and then *w*_*j*_ maps the vertex *a* to the vertex *v*_*i*_; that is, $v_{i}\overset{w_{i}}{\to }a\overset{w_{j}}{\to }v_{i}$. Since *w*_*i*_ is a composition of linear fractional transformations *x* and *y*, therefore *a* = *v*_*i*_. This implies that *γ* and *δ* are the same fragments, since a unique polynomial is obtained from a fragment. Hence, *F*[*w*_*i*_, *w*_*j*_] ~ *F*[*w*_*i*_, *w*_*i*_ *w*_*j*_].

Similarly, it is easy to prove that

(12) $$\begin{array}{c} F\left[w_{i},w_{j}\right]\sim F\left[w_{i},w_{i}w_{j}^{-1}\right],\;\;F\left[w_{i},w_{j}\right]\sim F\left[w_{i},w_{j}w_{i}\right],\\ F\left[w_{i},w_{j}\right]\sim F\left[w_{i},w_{j}w_{i}^{-1}\right]. \end{array}$$

Since ~ is an equivalence relation, hence

(13) F[wi,wj]~F[wi,wiwj]~F[wi,wiwj−1]~F[wi,wjwi]~F[wi,wjwi−1].

**Example 11 .**

**Figure 4:**
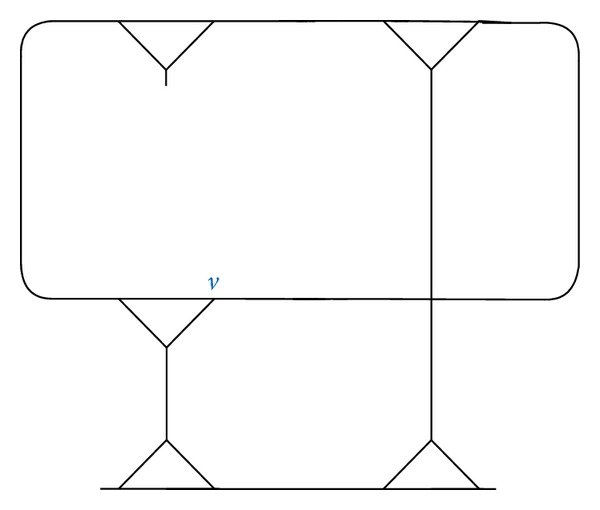


**Figure 5:**
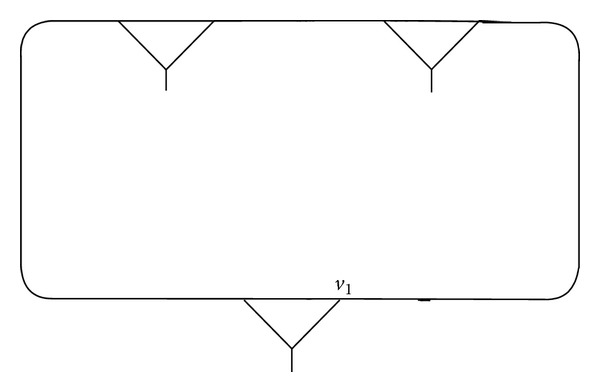


**Figure 6:**
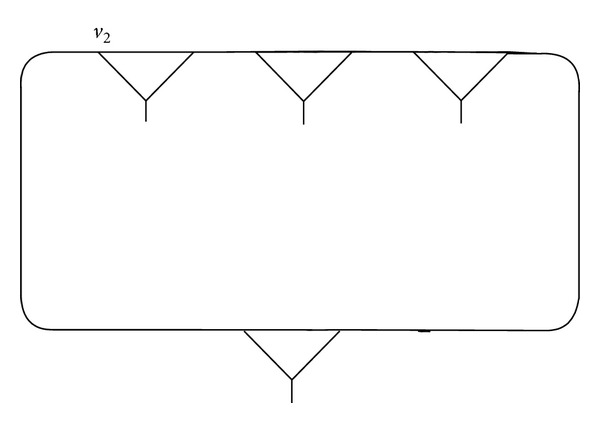


**Figure 7:**
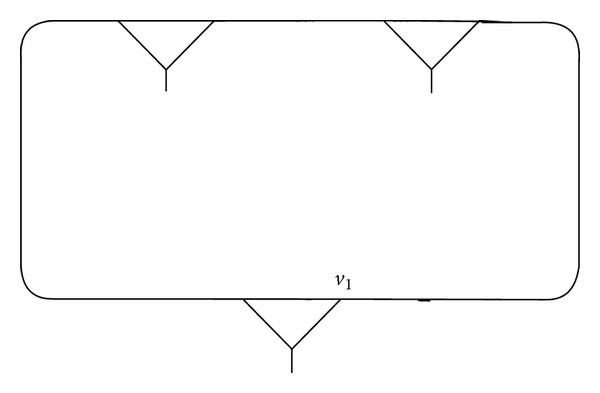


**Figure 8:**
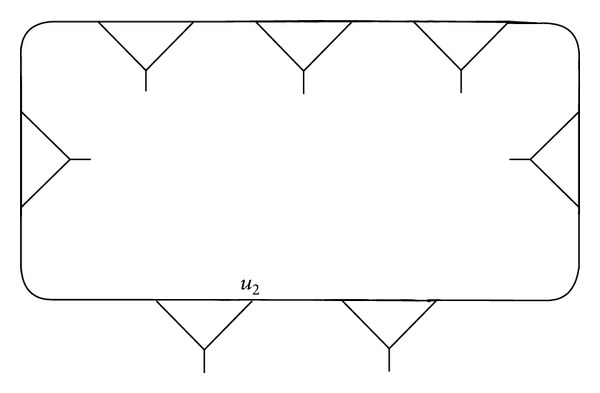


Theorem 10 shows that the fragment *γ*, which has a vertex fixed by *F*[*w*_*i*_, *w*_*j*_], and the fragment *δ*′, which has a vertex fixed by *F*[*w*_*i*_, *w*_*j*_ *w*_*i*_], are the same. So, the fragment, which has a vertex fixed by *F*[(*xy*)^2^(*xy* ^−1^), (*xy* ^−1^)(*xy*)^3^], and the fragment, which has a vertex fixed by *F*[(*xy*)^2^(*xy* ^−1^), (*xy* ^−1^)(*xy*)^5^(*xy* ^−1^)], are the same. Since the words (*xy*)^2^(*xy* ^−1^),  (*xy* ^−1^)(*xy*)^3^, and (*xy* ^−1^)(*xy*)^5^(*xy* ^−1^) correspond to the circuits (2,1), (3,1), and (5,2), so the fragment (Figure 4) is obtained, not only by joining the vertex *v*_1_ in (2,1) (Figure 5) with the vertex *v*_2_ in (3,1) (Figure 6) but also by joining the vertex *v*_1_ in (2,1) (Figure 7) with the vertex *u*_2_ in (5,2) (Figure 8).

**Theorem 12 .**

In a coset diagram for the action of *PGL*(2, *Z*) on *PL*(*F*_*q*_), if a vertex *v* is fixed by *w*, then the vertex (*v*)*t* is fixed by w*.

**Proof**

Let *w* = *xy* ^*η*_1_^ *xy* ^*η*_2_^ ⋯ *xy* ^*η*_*n*−1_^ *xy* ^*η*_*n*_^ be a word such that

(14) $$\begin{array}{c} \left(v\right)xy^{\eta_{1}}xy^{\eta_{2}}\cdots xy^{\eta_{n-1}}xy^{\eta_{n}}=v. \end{array}$$

Since (*xt*)^2^ = (*yt*)^2^ = 1, this implies that

(15) $$\begin{array}{c} yt=ty^{-1},\;\;y^{-1}t=ty,\;\;xt=tx. \end{array}$$

Now, by using (14) and (15), we have (*v*)*t* = ((*v*)*xy* ^*η*_1_^ *xy* ^*η*_2_^ ⋯ *xy* ^*η*_*n*−1_^ *xy* ^*η*_*n*_^)*t* = ((*v*)*t*)*xy* ^−*η*_1_^ *xy* ^−*η*_2_^ ⋯ *xy* ^−*η*_*n*−1_^ *xy* ^−*η*_*n*_^ = ((*v*)*t*)w*. This shows that *xy* ^−*η*_1_^ *xy* ^−*η*_2_^ ⋯ *xy* ^−*η*_*n*−1_^ *xy* ^−*η*_*n*_^ also fixes a point (*v*)*t*.

**Theorem 13 .**

If a vertex *v* is fixed by a word *w* in a circuit (*n*_1_, *n*_2_), then there exists a vertex v* in (*n*_1_, *n*_2_) such that v* is fixed by w*.

**Proof**

Consider a simple circuit (*n*_1_, *n*_2_).

**Figure 9:**
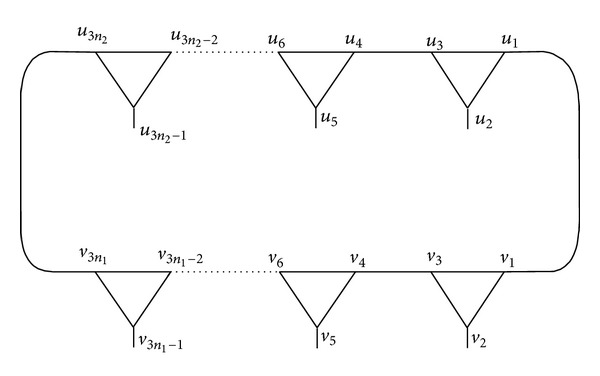


In Figure 9, one can see that the vertex *u*_1_ is fixed by (*xy*)^*n*_1_^(*xy* ^−1^)^*n*_2_^ and *u*_3*n*_2__ is fixed by (*xy* ^−1^)^*n*_1_^(*xy*)^*n*_2_^. So, *u*_3*n*_2__ = *u*_1_*. Also, the vertex *u*_2_ is fixed by *y*(*xy*)^*n*_1_^(*xy* ^−1^)^*n*_2_−1^ *xy* and *u*_3*n*_2_−1_ is fixed by *y* ^−1^(*xy* ^−1^)^*n*_1_^(*xy*)^*n*_2_−1^ *xy* ^−1^. So, *u*_3*n*_2_−1_ = *u*_2_*. Similarly, *u*_3*n*_2_−2_ = *u*_3_*, *u*_3*n*_2_−3_ = *u*_4_*,… .

The vertices *v*_1_ and *v*_3*n*_1__ are fixed by (*xy*)^*n*_2_^(*xy* ^−1^)^*n*_1_^ and (*xy* ^−1^)^*n*_2_^(*xy*)^*n*_1_^, respectively, implying that *v*_3*n*_2__ = *v*_1_*. Also, the vertices *v*_2_ and *v*_3*n*_1_−1_ are fixed by *y*(*xy*)^*n*_2_^(*xy* ^−1^)^*n*_1_−1^ *xy* and *y* ^−1^(*xy* ^−1^)^*n*_2_^(*xy*)^*n*_1_−1^ *xy* ^−1^, respectively, implying that *v*_3*n*_1_−1_ = *v*_2_*. Similarly, *v*_3*n*_1_−2_ = *v*_3_*, *v*_3*n*_2_−3_ = *v*_4_*,… .

**Figure 10:**
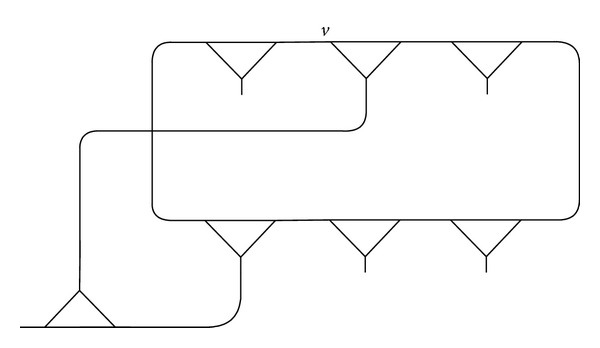


Let the homomorphic image of the fragment (Figure 10) occur in the coset diagram *D*(*θ*, *q*). Since *D*(*θ*, *q*) admits the axis of symmetry, the mirror image of the fragment under the permutation *t* will also occur as shown in Figure 11.

**Proposition 14 .**

If the fragment *γ* has one vertex *v* fixed by *F*[*w*_*i*_, *w*_*j*_], then its mirror image γ* has one vertex fixed by *F*[*w*_i_*, *w*_j_*].

**Proof**

Let *γ* be the fragment with one vertex *v* fixed by *F*[*w*_*i*_, *w*_*j*_]. Since γ* is the mirror image of *γ*, therefore γ* has one vertex (*v*)*t*. Then, by Theorem 12, the vertex (*v*)*t* is fixed by *F*[*w*_i_*, *w*_j_*].

**Theorem 15 .**

The polynomials obtained from the fragment *γ* and its mirror image γ* are the same.

**Proof**

Let *f*(*θ*) be the polynomial obtained by choosing a pair of words *F*[*w*_*i*_, *w*_*j*_] from *γ* and let *g*(*θ*) be the polynomial obtained by choosing a pair of words *F*[*w*_i_*, *w*_j_*] from γ*. Let *f*(*θ*) = 0. Then, by Theorem 2, there is a vertex say *v* in *D*(*θ*, *q*) or in $\bar{\text{PL}(F_{q})}$ which is fixed by *F*[*w*_*i*_, *w*_*j*_]. Then, by Theorem 12, the vertex (*v*)*t* is fixed by *F*[*w*_i_*, *w*_j_*]. So, by Theorem 2, *g*(*θ*) = 0. Similarly, it can be proved that if *g*(*θ*) = 0, then *f*(*θ*) = 0. Hence, *f*(*θ*) = *g*(*θ*).

Since a unique polynomial is obtained from a fragment *γ* and its mirror image γ*, hence *F*[*w*_*i*_, *w*_*j*_] ~ *F*[*w*_i_*, *w*_j_*].

Consider two circuits (*n*_1_, *n*_2_) and (*m*_1_, *m*_2_). In general, fragment *γ* and its mirror image γ* do not have the same orientation. There are certain fragments which have the same orientations as those of their mirror images. These kinds of fragments have vertical symmetry and may have fixed points of *t*. The following Theorem shows how these fragments are composed.

**Theorem 16 .**

(i) The fragment composed by joining a vertex *u*_1_, fixed by

(16) $$\begin{array}{c} {\left(xy^{-1}\right)}^{\left(n_{1}-1\right)/2}{\left(xy\right)}^{n_{2}}{\left(xy^{-1}\right)}^{\left(n_{1}+1\right)/2} \end{array}$$

in (*n*_1_, *n*_2_), with the vertex *u*_2_, fixed by (*xy* ^−1^)^(*m*_1_ − 1)/2^(*xy*)^*m*_2_^(*xy* ^−1^)^(*m*_1_ + 1)/2^ in (*m*_1_, *m*_2_), has the same orientation as that of its mirror image.

(ii) The fragment composed by joining a vertex *u*_1_, fixed by

(17) $$\begin{array}{c} {\left(xy^{-1}\right)}^{n_{1}/2}{\left(xy\right)}^{n_{2}}{\left(xy^{-1}\right)}^{n_{1}/2} \end{array}$$

in (*n*_1_, *n*_2_), with the vertex *u*_2_, fixed by (*xy* ^−1^)^*m*_1_/2^(*xy*)^*m*_2_^(*xy* ^−1^)^*m*_1_/2^ in (*m*_1_, *m*_2_), has the same orientation as that of its mirror image.

(iii) The fragment composed by joining a vertex *v*_1_, fixed by

(18) $$\begin{array}{c} {\left(xy^{-1}\right)}^{n_{1}/2}{\left(xy\right)}^{n_{2}}{\left(xy^{-1}\right)}^{n_{1}/2} \end{array}$$

in (*n*_1_, *n*_2_), with the vertex *v*_2_, fixed by (*xy*)^*m*_1_/2^(*xy* ^−1^)^*m*_2_^(*xy*)^*m*_1_/2^ in (*m*_1_, *m*_2_), has the same orientation as that of its mirror image.

**Proof**

(i) Let *γ*_1_ be the fragment which has one vertex say *u*, fixed by

(19) $$\begin{array}{c} {\left(xy^{-1}\right)}^{(n_{1}-1)/2}{\left(xy\right)}^{n_{2}}{\left(xy^{-1}\right)}^{(n_{1}+1)/2},\\ {\left(xy^{-1}\right)}^{(m_{1}-1)/2}{\left(xy\right)}^{m_{2}}{\left(xy^{-1}\right)}^{(m_{1}+1)/2}. \end{array}$$

Then, by Proposition 14, its mirror image fragment *γ*_1_* contains a vertex, say, u*, fixed by (*xy*)^(*n*_1_−1)/2^(*xy* ^−1^)^*n*_2_^(*xy*)^(*n*_1_+1)/2^ and (*xy*)^(*m*_1_−1)/2^(*xy* ^−1^)^*m*_2_^(*xy*)^(*m*_1_+1)/2^. By Remark 4, the vertex (u*)*y* of fragment *γ*_1_* is fixed by

(20) y(xy)(n1−1)/2(xy−1)n2(xy)(n1+1)/2y−1 =((xy−1)(n1−1)/2(xy)n2(xy−1)(n1+1)/2)−1

and *y*(*xy*)^(*m*_1_−1)/2^(*xy* ^−1^)^*m*_2_^(*xy*)^(*m*_1_+1)/2^ *y* ^−1^ = ((*xy* ^−1^)^(*m*_1_−1)/2^(*xy*)^*m*_2_^(*xy* ^−1^)^(*m*_1_+1)/2^)^−1^. This implies that *u* = (u*)*y*. Hence fragment *γ*_1_ has the same orientation as that of its mirror image *γ*_1_*.

(ii) Let *γ*_2_ be the fragment which has one vertex, say, *v*, fixed by

(21) $$\begin{array}{c} {\left(xy^{-1}\right)}^{n_{1}/2}{\left(xy\right)}^{n_{2}}{\left(xy^{-1}\right)}^{n_{1}/2},\\ {\left(xy^{-1}\right)}^{m_{1}/2}{\left(xy\right)}^{m_{2}}{\left(xy^{-1}\right)}^{m_{1}/2}. \end{array}$$

Then, its mirror image fragment *γ*_2_* has one vertex, say, v*, fixed by

(22) $$\begin{array}{c} {\left(xy\right)}^{n_{1}/2}{\left(xy^{-1}\right)}^{n_{2}}{\left(xy\right)}^{n_{1}/2},\;\;{\left(xy\right)}^{m_{1}/2}{\left(xy^{-1}\right)}^{m_{2}}{\left(xy\right)}^{m_{1}/2}. \end{array}$$

The vertex (v*)*x* of fragment *γ*_2_* is fixed by

(23) x(xy)n1/2(xy−1)n2(xy)n1/2x =((xy−1)n1/2(xy)n2(xy−1)n1/2)−1

and *x*(*xy*)^*m*_1_/2^(*xy*)^*m*_2_^(*xy*)^*m*_1_/2^ *x* = ((*xy* ^−1^)^*m*_1_/2^(*xy*)^*m*_2_^(*xy* ^−1^)^*m*_1_/2^)^−1^. This implies that *v* = (v*)*x*. Hence, *γ*_2_ has the same orientation as that of its mirror image *γ*_2_*.

(iii) Let *γ*_3_ be the fragment which has one vertex, say, *v*, fixed by

(24) $$\begin{array}{c} {\left(xy^{-1}\right)}^{n_{1}/2}{\left(xy\right)}^{n_{2}}{\left(xy^{-1}\right)}^{n_{1}/2},\;\;{\left(xy\right)}^{m_{1}/2}{\left(xy^{-1}\right)}^{m_{2}}{\left(xy\right)}^{m_{1}/2}. \end{array}$$

Then, its mirror image fragment *γ*_3_* has one vertex, say, v*, fixed by

(25) $$\begin{array}{c} {\left(xy\right)}^{n_{1}/2}{\left(xy^{-1}\right)}^{n_{2}}{\left(xy\right)}^{n_{1}/2},\;\;{\left(xy^{-1}\right)}^{m_{1}/2}{\left(xy\right)}^{m_{2}}{\left(xy^{-1}\right)}^{m_{1}/2}. \end{array}$$

The vertex (v*)*x* of fragment *γ*_3_* is fixed by

(26) x(xy)n1/2(xy−1)n2(xy)n1/2x =((xy−1)n1/2(xy)n2(xy−1)n1/2)−1

and *x*(*xy* ^−1^)^*m*_1_/2^(*xy*)^*m*_2_^(*xy* ^−1^)^*m*_1_/2^ *x* = ((*xy*)^*m*_1_/2^(*xy* ^−1^)^*m*_2_^(*xy*)^*m*_1_/2^)^−1^. This implies that *v* = (v*)*x*. Hence, *γ*_3_ has the same orientation as that of its mirror image *γ*_3_*.

## 4. Motivation and Open Problems

If we join a pair of circuits at a certain point, we get a fragment and hence a polynomial. Since each such polynomial splits linearly in a suitable Galois [16] and corresponding to each zero, we get a triplet $\left(\bar{x},\bar{y},\bar{t}\right)$ [15] which is a group. This shows that each pair of circuits can be related to a group. A pair of circuits has finitely many points of connections. Two distinct points of connection may or may not give the same fragment. So, it is important to ask the following question.

**Problem 17 .**

How many fragments (polynomials) are obtained if we join a pair of circuits (*n*_1_, *n*_2_) and (*m*_1_, *m*_2_) at all points of connection?

In order to give the answer of the above question, we first have to find those pair of words which are equivalent. In other words, we have to find those points of connection, at which we get the same polynomial. This issue is resolved in this paper. We will give the answer of the above question in an other paper. After that, one can establish a connection between a class of groups and a pair of circuits (*n*_1_, *n*_2_) and (*m*_1_, *m*_2_), which is indeed a great development.

## Figures and Tables

**Figure 11:**